# Chinese characters reveal impacts of prior experience on very early stages of perception

**DOI:** 10.1186/1471-2202-12-14

**Published:** 2011-01-26

**Authors:** Tobias Elze, Chen Song, Rainer Stollhoff, Jürgen Jost

**Affiliations:** 1 Max Planck Institute for Mathematics in the Sciences, Inselstr. 22,04103 Leipzig, Germany; 2 Institute of Cognitive Neuroscience, University College London, London WC1N 3AR, UK; 3 Santa Fe Institute, 1399 Hyde Park Road, Santa Fe, NM 87501, USA

## Abstract

**Background:**

Visual perception is strongly determined by accumulated experience with the world, which has been shown for shape, color, and position perception, in the field of visuomotor learning, and in neural computation. In addition, visual perception is tuned to statistics of natural scenes. Such prior experience is modulated by neuronal top-down control the temporal properties of which had been subject to recent studies. Here, we deal with these temporal properties and address the question how early in time accumulated past experience can modulate visual perception.

**Results:**

We performed stimulus discrimination experiments and compared a group of Chinese participants with a German control group. The perception of our briefly presented visual objects (targets) was disturbed by masking stimuli which appeared in close spatiotemporal proximity. These masking stimuli were either intact or scrambled Chinese characters and did not overlap with the targets. In contrast to German controls, Chinese participants show substantial performance differences for real versus scrambled Chinese characters if these masking stimuli were presented as early as less than 100 milliseconds after the onset of the target. For Chinese observers, it even occured that meaningful masking stimuli enhanced target identification if they were shown at least 100 milliseconds after target onset while the same stimuli impaired recognition if presented in close temporal proximity to the target. The latter finding challenges interpretations of our data that solely rely on stimulus contours or geometric properties and emphasizes the impact of prior experience on the very early temporal dynamics of the visual system.

**Conclusions:**

Our findings demonstrate that prior experience which had been accummulated long before the experiments can modulate the time course of perception intriguingly early, namely already immediately after the perceptual onset of a visual event. This modulation cannot solely operate as a feedback in response to the visual event but is rather a permanent effect.

## Background

The optimal application of accumulated past experience about the visual world is considered a key property of perception. Already in the 19th century, von Helmholtz [1] introduced the concept of "unconscious inference" which explains visual perception as an interaction of experience-guided unconscious hypotheses about the sensory input and the sensory input itself. In recent years, this inference concept has become increasingly popular in vision science. Popular recent theoretical frameworks with focus on such perceptual prior experience are, for instance, Bayesian decision theory [2,3] and empirical ranking theory [4,5]. The perceptual inference concept has been suggested in many different areas, such as classification [6], object perception [7], the analysis of natural environments [8], and neuronal computation [9-11].

Here, we regard inference as an interplay between lower and higher cortical areas that works as follows: Primary sensory cortical areas receive bottom up input from the sensors and simultaneously top down input from higher areas which transmit neuronal predictions of perceptual hypotheses. In an iterative process, these hypotheses evolve over time and modulate the neuronal representations at the lower areas until a final decision about the input is reached. This adjustment process of top down predictions and bottom up evidence is subject to prior expectations originating from the observer's experience over lifetime. There are numerous examples that perception is strongly determined by accumulated experience with the world. For instance, prior knowledge that light usually comes from above has been shown to influence shape perception [12], knowledge about the natural color of fruits modulates the color appearance of objects [13], position perceptions of moving objects are determined by accumulated experience with image speeds [14], or visual perception is tuned to statistics of natural scenes, e.g. spectral statistics of natural scences predicts perceptual color qualities [15] or experience with object positions in natural scenes constitutes perceptual priors which modulate the locations of observers' first saccades [16]. In addition, inference can be examined in behavioral studies. It could be shown, for instance, that observers combine visual featural information with externally assigned values to stimuli in a way that is consistent with optimal Bayesian inference [17], or influences of experience have been demonstrated to change size-weight priors [18]. Furthermore, experimental findings from brain imaging that predictable visual input is processed with less neural activation in the primary visual cortex [19] give additional evidence for the concept of perceptual priors, and the cortical facilitation for emotionally arousing and therefore highly meaningful stimuli has been shown to be rapid [20].

While it is commonly accepted that such perceptual priors play an important role in higher level visual processing, many phenomena in low level vision are explained in terms of purely feedforward information flow which is not influenced by the observers' prior experience. Top down influences in visual experiments are often restricted to the immediate spatial context, or perceptual priors are built up immediately before the observers' decisions (visual priming experiments) but are not acquired over the observers' lifetime. Here, we investigate the influences of accumulated perceptual prior experience on the very early temporal course of visual perception by the psychophysical experimental paradigm Object Substitution Masking [21] (OSM). In OSM, a target stimulus is flashed for a duration of around 10 milliseconds. A second stimulus, the mask, is presented in close spatiotemporal proximity to the target. Although both stimuli are non-overlapping, the mask impairs target identification performance. Usually, the mask impact is controlled by either the mask duration or, as in the current study, the temporal interval between target and mask called interstimulus interval (ISI).

In OSM, the impairment of target visibility is explained as the disturbance of the inference between higher cortical hypotheses and lower area visual input [22,23]. In contrast to low level feedforward explanations for many other perceptual impairment phenomena, the OSM theory assumes that target and mask do not compete at the level of stimulus configurations or contours but rather on an object level. For certain ISIs, the temporal dynamics of the perceptual inference cause the inhibition of the "target hypothesis" by the "mask hypothesis". Masking is thus caused by the substitution of the target object with the mask object. The iterative inference process in the OSM concept does not take perceptual priors into account. If the target identification impairment is an indicator for very early visual inference, as suggested by the OSM model, we expect to see an influence of perceptual priors. Although the target impairment effect occurs as early as within the first 100 ms after the perceptual onset of the target, groups of observers with different priors should reveal differences in their OSM time course even if exposed to identical target and mask stimuli.

**Figure 1:**
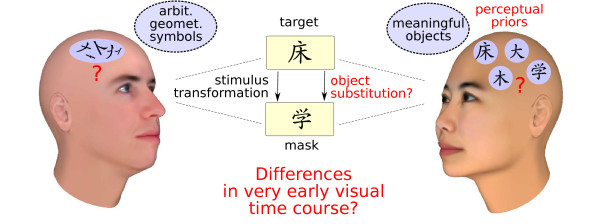
**Rationale of the experiment**. Chinese and German participants have different perceptual priors in a backward masking task with Chinese characters. While for German observers, one arbitrary geometrical object is followed by another, Chinese participants will perceive an object substitution of a meaningful target object by a meaningful mask object. Will the different priors have an influence on the visual time course already immediately after the ultrashort stimulus presentations?

In order to test this hypothesis, we performed OSM experiments using Chinese characters as stimuli and compared the results of a group of native Chinese observers to a German control group. Figure 1 outlines our rationale: For German observers, these characters are supposed to be arbitrary geometrical symbols. If a Chinese target character is followed by a Chinese character mask, this means nothing more than the transformation of one meaningless geometrical symbol to another. For Chinese participants, Chinese characters are meaningful objects to which they were frequently exposed over their lifetime. If a Chinese character target is followed by another Chinese character, two hypotheses that are strongly preactivated due to character priors, the hypothesis of the target character and that of the mask character, will compete in the inference process. Object substitution takes place between two stimuli of an equal degree of "objectness", and we expect a higher masking magnitude compared to a mask which is not a Chinese character. Therefore, the strong familiarity with the mask will impede the correct identification of the target. For German participants, we expect no differences between Chinese character masks and other masks.

As shown already a decade ago [23], the target impairment effect of OSM can be obtained by such simple masking stimuli as four dots surrounding the target, and the strength of the effect is modulated by attentional factors auch as set size. DiLollo and colleagues [23] systematically varied set sizes and demonstrated strong effects for sizes of eight stimuli or more. Jiang and Chun [24] received OSM effects even when target and mask stimuli were spatially clearly separated. In their experiments, they presented eight stimuli in a circular manner. We used the same spatial configuration in our experiments, as detailed below.

**Figure 2:**
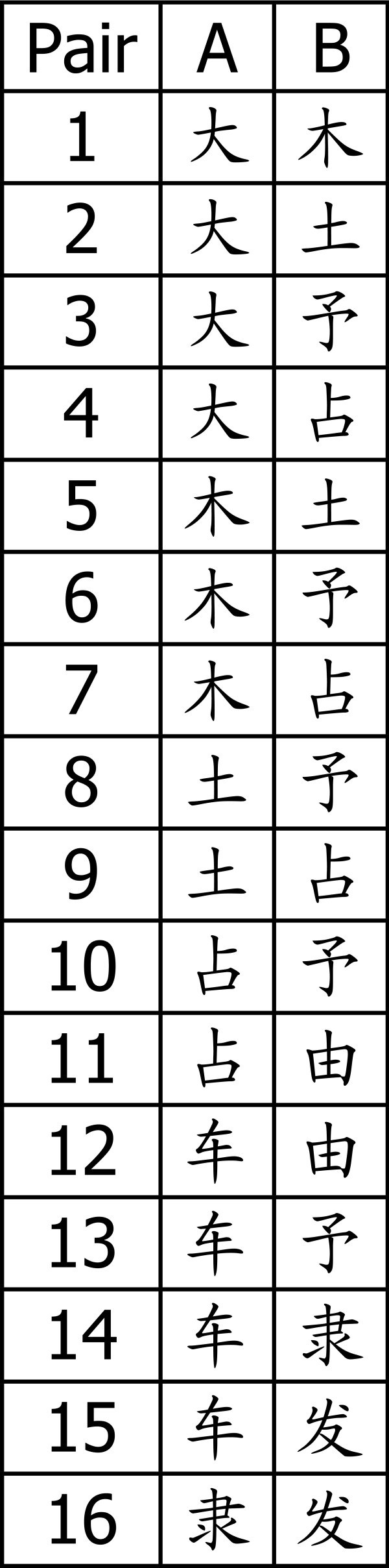
**Target stimuli**. The 16 pairs of Chinese characters used in the discrimination task. This table was visible to the participants throughout the whole experiment.

Our experiments consisted of 16 subsessions in each of which observers had to report which character of a given pair of characters had been presented. The 16 pairs are shown in Figure 2. The respective pair for each subsession was chosen in random order.

**Figure 3:**
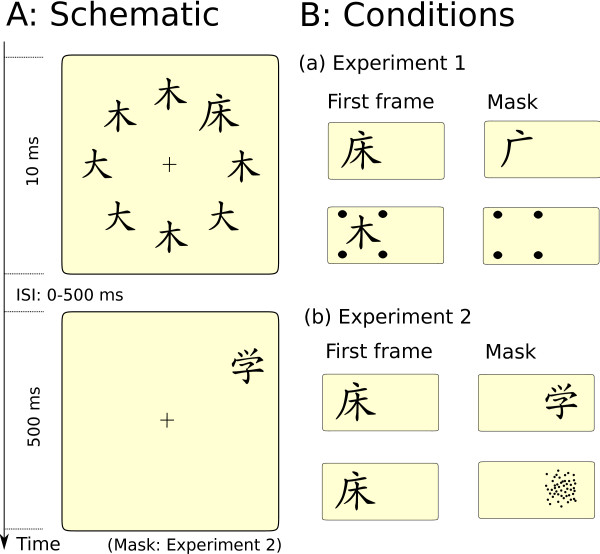
**Experimental setup**. A: Schematic of the temporal course of the two experiments. A circular display of eight stimuli is flashed for 10 ms. One stimulus, the target, is indicated. After a blank interstimulus interval (ISI), a second stimulus, the mask, is shown for half a second (here, the intact mask of Experiment 2 is shown). B: The conditions of the two experiments: In the first frame of both experiments, the target is indicated either by a Chinese character or by four dots. In Experiment 1, the indicator reappears as the mask (a). In Experiment 2, the mask is another Chinese character (either intact or scrambled), shifted to the position right next to where the the target had appeared before (b).

We presented our target stimuli together with distrators in the following way: In each experimental trial of each subsession, eight characters of the respective pair were flashed in a circular arrangement. Figure 3A shows an example for pair 1. The randomly chosen target position was indicated in half of the trials by a surrounding character, chosen such that the combination of the two characters made up another Chinese character (the north-east position in Figure 3A shows the resulting combination). In the other half of the trials the indicator stimulus consisted of four surrounding dots.

After a blank ISI of variable length (see Methods), a mask stimulus appeared at a position immediately right to the target for 500 ms. Figure 3B shows the different masks used in the two experiments. In Experiment 1, the mask was identical to the indicator stimulus (a), whereas in Experiment 2, the mask was a different stimulus which appeared next to the target position (b). In half of the cases, the mask of Experiment 2 was an intact Chinese character, in the other half a scrambled version of this character. The observers were instructed to ignore the mask. After the mask offset, the participant had to report in a two alternative forced choice task which of the two characters of the pair had been shown as target character by pressing either the left or the right mouse button.

## Results and Discussion

We represent participants' correctness for each condition by expectation values *E*. Details about the calculations of *E *and the further data analyses can be found in the Methods section. The results for all 16 pairs were averaged for each observer.

**Figure 4:**
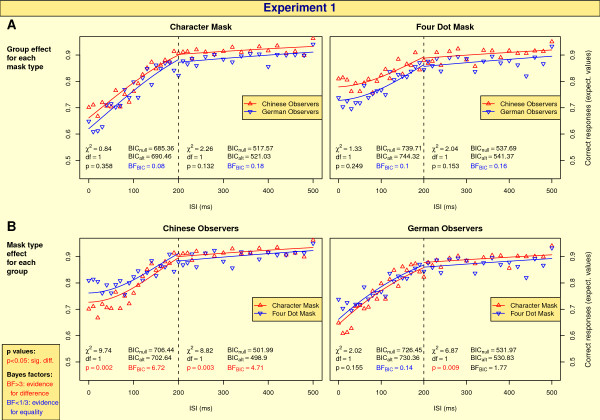
**Results of Experiment 1**. Group effect for each mask type (A) and mask type effect for each experimental group (B). The triangular symbols denote means over the observers which were calcultated after a logit transform. The vertical dashed line denotes the separation between short (≤ 200 ms) and long ISIs. Differences for short ISIs give evidence for mask influences, differences for long ISIs reflect target effects. The two solid lines show the GLM model fits for each condition. The statistical measures of two independent tests (χ^2 ^test and BIC Bayes factor approximation, see text for details) are given below the plots, separately for short and long ISIs.

**Figure 5:**
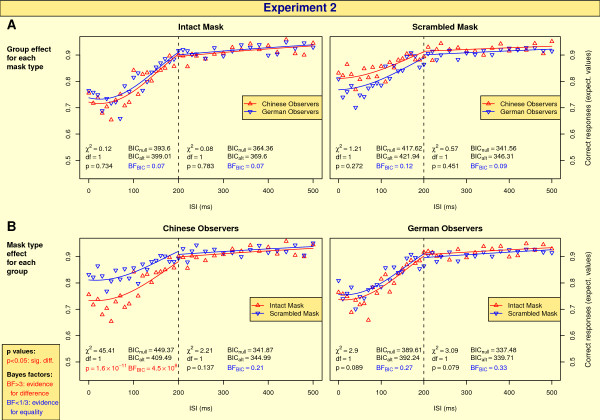
**Results of Experiment 2**. See caption of Fig. 4 for details.

ISIs for which the mask follows shortly after the target are subject to relatively strong mask influences. From a certain ISI on, target and mask are temporally so distant that the influence of the mask will vanish. Further increases of ISI durations will keep the correct identification responses constant, so that condition differences for such ISIs cannot be attributed to the mask but need to be target effects. A visual inspection of the results (Figure 4 and 5) indicates such a saturation of performance for ISIs from around 200 ms onwards. Therefore, we analyzed the correct responses for ISIs ≤ 200 ms and ISIs > 200 ms separately.

In order to test for group and mask type effects, we fitted models to the data. Details about the models can be found in the Methods section. The solid lines in Figure 4 and Figure 5 show the optimal model fits for each condition and illustrate which of the two respective conditions yields better performance.

We performed two independent statistical tests to analyze evidence for the Null hypothesis *H*_0 _of equality and the alternative hypothesis *H*_1 _of non-equality: First, we calculated a likelihood ratio χ ^2 ^test (in the following "χ^2 ^test"). Second, we approximated the Bayes Factor (BF; see, for instance, [25-27]) of the models by the Bayesian information criterion (BIC) [28]. See Methods for further details.

Following statistical conventions, we call a difference "significant" if its χ^2 ^test *p *value is less than 0.05. Furthermore, we infer a positive evidence for the difference (*H*_1_) for a Bayes factor (BF) greater than 3 and a positive evidence for equality (*H*_0_) for $\text{BF}<\frac{1}{3}$. A BF inside the interval $\left[\frac{1}{3},3\right]$ reflects statistical uncertainty about which of the hypotheses is correct. Note that the χ^2 ^*p *value is the probability for the data assuming *H*_0 _is true. It can be used to reject *H*_0 _but in contrast to the BF it is uninformative about evidence in favor of *H*_0_.

### Results of Experiment 1

Figure 4A and 4B show summarizing statistics of the mask type effect for each group and the group effect for each mask type, respectively. In addition, the results of the single observers are shown in Fig. S1 in "Supplementary Figures" (see Additional file 1).

#### Initial training

Chinese observers needed on average 12.5 trials (standard deviation: 7.6) to pass the training. German observers needed 19.4 trials (std: 13.9). A two sample Wilcoxon test [29] did not yield significant group differences.

#### Group effects

The solid lines in Figure 4A indicate a trend that Chinese observers perform better than German observers for both mask types. However, this trend is not significant and the Bayes factors give positive evidence for *H*_0_.

#### Mask type effects

For long ISIs, Chinese participants perform significantly better for character masks (both tests favor *H*_1_). For German observers, the χ^2 ^test yields a significantly better performance for long ISIs and character masks as well, but the Bayes factor (1.77) shows at most a trend but is still in the uncertainty interval $\left[\frac{1}{3},3\right]$.

For short ISIs, Chinese observers perform significantly better for four dot masks, in accordance with the Bayes factor, whereas for German observers, *H*_0 _is favored.

### Results of Experiment 2

Figure 5A and 5B show summarizing statistics of the mask type effect for each group and the group effect for each mask type, respectively. In addition, the results of the single observers are shown in Fig. S2 in "Supplementary Figures" (see Additional file 1).

#### Initial training

Chinese observers needed on average 11.0 trials (standard deviation: 2.6) to pass the training. German observers needed 18.4 trials (std: 11.14). A two sample Wilcoxon test [29] did not yield significant group differences.

#### Group effects

There is no significant group effect for any of the mask types, and the Bayes factors favor *H*_0 _(see Figure 5A).

#### Mask type effects

For long ISIs, there is no significant mask type effect for any of the groups (see Figure 5B). For short ISIs, Chinese participants perform significantly better for scrambled masks, and the Bayes factor gives very strong evidence for *H*_1 _accordingly. For German observers, there is no significant difference, and the Bayes factor gives evidence for *H*_0_.

#### Indicator effects

**Figure 6:**
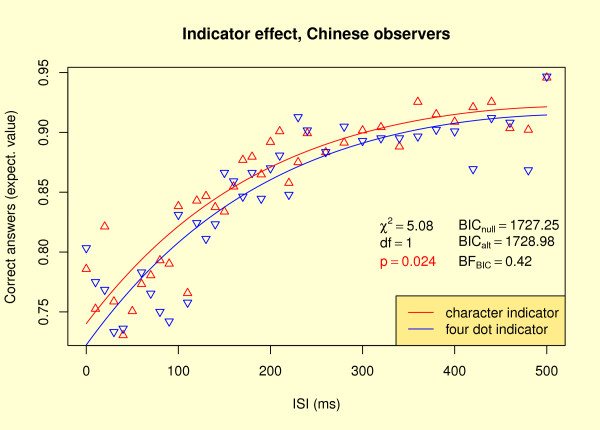
**Indicator effect**. Indicator effect for Experiment 2, Chinese observers. See caption of Fig. 4 for details about the statistics.

As Experiment 2 separates indicator and mask, we analyzed the indicator effects (character vs. four dots) over the whole range of ISIs (see Methods for details). Whereas for German observers none of the tests favored an indicator difference (χ^2 ^= 0.50, *df *= 1, *p *= 0.48; BIC(null) = 1582.86, BIC(alt) = 1589.18, BF_BIC _= 0.0425), the χ^2 ^test for Chinese participants is significant and masking seems to be stronger for four dot indicators, as shown in Figure 6. However, the corresponding Bayes factor (0.42) is inside the interval of uncertainty ($\left[\frac{1}{3},3\right]$).

### Target effects

Chinese characters are meaningful and frequently encountered objects for native Chinese, therefore one might expect that the ability to discriminate between two Chinese characters is generally higher for Chinese observers compared to German controls, independent of masking. In our experiments, this expectation could not be confirmed: At most, the line plots in Figure 4A for both mask types and in Figure 5A for scrambled masks indicate a trend of better performance of Chinese participants, but our statistics give evidence for *H*_0 _in all these cases. That is, Chinese observers do not benefit from the object properties of the targets. Pure target category effects (meaningful vs. meaningless) do not play a role in our experiments. Not that although a visual inspection of group differences in performance (Figure 4A) leads to the impression of significance, group differences in mean are masked by large variability across observers within each group. In contrast to tests for group differences, this inter-observer variability only has a minor influence on tests of within-observer differences, e.g. mask type effects (see below).

### Mask and indicator effects

Mask effects can be inferred from the short ISI analyses in Figure 4B and Figure 5B. In accordance with our assumption that prior experience can modulate the early time course of visual perception, for German observers it did not influence performance whether the mask was a Chinese character or a meaningless symbol, whereas the mask type impact for Chinese participants was substantial in both experiments. Chinese observers are significantly more impaired by real Chinese character masks, even if the latter appear at a spatially different location (as in Exp. 2). This provides evidence for our speculation illustrated in Figure 1 that for Chinese participants an object substitution between target and mask occured.

Indicator effects can be analyzed by focussing on long ISIs in Figure 4B: If the ISI is long enough, the mask impact on target discrimination will be weak, so performance differences can be attributed to the efficiency of the indicators in the first presentation frame (see Figure 3). As masks and indicators were identical in Exp. 1, long ISI differences are likely to represent pure indicator effects in Figure 4B.

For Chinese participants, there is a substantial advantage for character indicators. This result is not surprising as character indicators together with the targets shape new real Chinese characters whereas four dot indicators slightly weaken the perception of "objectness" of the targets. For German observers, there seems to be the same trend, but only one of our two statistical tests favors a character indicator advantage whereas the other test yields uncertainty about the decision whether to prefer *H*_0 _or *H*_1_. This trend might originate from the higher energy of the character indicator compared to the four dots so that the former has a higher impact on guiding attention to the target among the distractors.

Exp. 2 separates masks from indicators. If there had been an indicator effect independent of masking for Chinese observers in Exp. 1, we would expect such an effect for all ISIs in Exp. 2 as well. Figure 6 indicates at least an according trend: While one of our two tests supports an advantage for character indicators, the second test reports uncertainty. For German observers, we did not find an indicator effect in Exp. 2. Taken together, the substantial character indicator advantage in Exp. 1 and the according trend in Exp. 2 for Chinese participants give evidence that two pure Chinese characters are easier to discriminate for Chinese observers than two Chinese characters that are degraded by meaningless surrounding symbols (four dots).

### Evidence for perceptual inference

Our results suggest an influence of perceptual inference on very early temporal processing: First, as in [24], we find that target and mask stimuli can be spatially clearly separated (Figure 3B(b)) so that contour similarity effects as a cause of masking are unlikely. Instead, target and mask are likely to be processed as objects, and the concept of OSM provides an explanation for the masking effect within each group, although it alone cannot explain the group differences between Chinese and Germans.

Second, as we presented the same stimuli to both groups, spatial arrangement or contour theories cannot account for the substantial differences for Chinese in contrast to German participants.

Third, the interaction effect for Chinese observers in Figure 4B is of special interest: For long ISIs, i.e. when mask impact is weak, it is easier to discriminate pure Chinese characters than Chinese characters surrounded by four dots. This is compatible with previous findings that target stimuli with greater meaning are less vulnerable to masking, which has been shown for targets like words or faces before [30]. However, if parts of the targets become masks, this effect is reversed, as can be seen for short ISIs. The greater meaning of the mask interacts with the greater meaning of the target and reverses the target meaning advantage. These findings support an inference explanation, that is the disruption of a reasoning process in which competing hypotheses are checked which are either from equally strong or from differently strong object categories.

Not only this interaction provides evidence for an inference process but also the nature of the difference in masking found for Chinese participants: Masking is stronger for character masks compared to non-character masks. For scrambled or four dot masks, a Chinese character, that is a sensible and frequently encountered object, is followed by an unintelligible symbol, whereas for character masks, the target is followed by another stimulus with the same "object qualities" which leads to object substitution within the same category (see Figure 1). As hypothesized above, the high degree of familiarity with the mask impedes the target discrimination for Chinese participants.

### Object categorization speed and time course

Our study deals with rapid forced choice decisions between two object alternatives. Our results might be of interest for a different but related field: In recent years, a number of studies investigated the remarkable speed of visual object categorization [31] which was characterized by electrophysiological differences or saccade direction differences that could be recorded already 150 ms and earlier after the stimulus onset [32,33]. In such a two alternative forced choice task, the perceptual system can tune its prior projections based on experience. Such focused top-down projections might be the reason for early differences in electrophysiological and behavioral measurements as well as for the very early masking curve differences found in our study.

Brain imaging studies about the temporal sequence of inference processes are rare due to poor temporal resolution of common functional brain imaging techniques. Two recent studies focussed on the time course of attentional control by combining of electrophysiology and neuroimaging [34,35] and discussed contributions of frontal and parietal brain areas. Visuospatial attention as a mechanism of top down modulation of object perception might be closely related to the effects described in the current study. Our results and our experimental paradigm are hoped to inspire similar multi-methodological approaches to neuroanatomy which focus on prior experience on object recognition and reveal the temporal sequences of inference in the brain.

## Conclusions

Previous experiments gave rise to the explanation that hypotheses about percepts are iteratively checked against the visual input [22] and are therefore compatible with the idea of perceptual inference. Our results add an important further evidence for the inference concept as it shows that the very early perceptual phenomenon OSM can be modulated by perceptual priors.

Our study demonstrates therefore an intriguingly early influence of accumulated prior experience that was built up independently of and long before our experiments. This influence of prior experience occurs as early as between 0 and 100-200 milliseconds after the perceptual onset of a visual event.

## Methods

The study is in compliance with the Helsinki Declaration http://www.wma.net/en/30publications/10policies/b3/index.html and has been approved by the local ethics committee of the Max Planck Institute for Mathematics in the Sciences (reference key: ElzeT1).

### Participants

The participants were recruited via a notice posted on campus of Leipzig University and were either master or PhD students or visiting guests. They were paid 7.5 Euro per hour for participation. All participants gave written informed consent to participate in the study.

Chinese observers were Chinese native speakers who had lived for no longer than six years in Germany. German observers reported no knowledge of Chinese language. All participants were naive w.r.t. the experiment and had normal or corrected to normal sight.

Participants in Experiment 1 were nine Chinese observers (3 female, 6 male, age range: 23 to 30, median age: 26) and nine German observers (5 female, 4 male, age range: 23 to 39, median age: 29). Experiment 2 has been conducted 2 months after Experiment 1 and was intended to confirm the effect found in Experiment 1 and to investigate further details of the findings. We contacted the observers who had participated in Experiment 1. Six of the German observers could participate in Experiment 2 (3 female, 3 male, age range: 26 to 39, median age: 29.5). Only 3 of the Chinese participants were still available for Experiment 2. In order to match the size of the groups, we found 3 additional Chinese observers via another notice on campus. The age range of the Chinese participants was 22 to 28 (1 female, 5 male, median age: 26). As the effect found in Experiment 1 had been substantial, we found it justified to perform Experiment 2 with six participants per group in contrast to the nine observers who had participated in Experiment 1.

### Apparatus

Stimuli were shown on an IIYAMA VisionMasterPro 514 monitor at a resolution of 800 × 600 and a refresh rate of 200 Hz (frame duration: 5 ms). The experiments were controlled by the program FlashDot which was especially developed for this purpose and which we provide as an open source implementation for free http://www.flashdot.info. Presentation timing specifications in text and figures were calculated by (number of frames) × (frame duration). Note that this method of timing specification, although frequently used, neglects the effect of CRT phosphor decay [36].

### Stimuli and Procedure

The experiments consisted of 16 subsessions in each of which observers had to perform a two alternative forced choice task by reporting which character of a given pair of characters had been presented. The 16 pairs are shown in Figure 2. The respective pair for each subsession was chosen in random order. The participant was instructed to report which of the two characters had been shown in each of the forthcoming trials by pressing either the left or the right mouse button.

All stimuli were presented on a black background. The Chinese character stimuli were centered on invisible squares with a width of 2.29 visual degree. On any given trial, the circular display (radius: 3.57 degree) of the eight Chinese characters was flashed for 2 frames. One of the eight characters was indicated to be the target, in half of the cases by a surrounding character, in the other half by four dots. Observers fixated centrally.

After the disappearance of the circular display and a blank ISI lasting *q *∈ {0, 2, 4, ... 48, 52, 56, 60, ... 100} frames, the mask was shown on the right hand side of the indicated target for 100 frames. In Experiment 1, the mask was identical to the indicator. In Experiment 2, in 50% of the cases, the mask was a Chinese character different from the target pair, in all other cases a scrambled version, where scrambling was realized by shuffling the pixel matrix of the character. Moreover, in half of the cases the indicator was a character as above, in the other half four dots. There were 16 subsets of trials determined by the 16 target pairs.

After the disappearance of the trailing mask, a blank screen indicated the participant to give the response. Observers were instructed to report as correctly as possible. Each ISI *q *occured four times in each trial subset in random order, so that each observer gave 16 (pairs) × 38 (ISIs) × 4 (repetitions) = 2432 responses. Between the trials, a central fixation cross was shown that disappeard one frame before the start of the next trial.

Prior to each experiment, each participant underwent a training in order to get familiar with the experimental tasks. In the training, the circular display for pair 1 was flashed together with the four dot indicator, but no mask was shown (pure target discrimination task). The training was finished when the participant answered correctly in more than 80% of the preceding 10 trials. A participant could pass the training with a minimum number of nine trials (in the case that the participant responded to all of them correctly).

### Data Analysis

#### Models

We represent observers' correctness for each condition by expectation values *E *calculated according to the Laplace Rule of Succession ($E=\frac{s+1}{n+2}$, *s*: number of successes, *n*: number of all trials in the condition). For further analyses, we applied a Generalized Linear Model approach and linearized the *E *values to *y *values by the logit transform: $y=\log\text{it}\left(E\right)=\log\left(\frac{E}{1-E}\right)$.

In order to analyze the effects of the respective conditions (mask type/group/indicator), we fitted models to the logits *y*. We analyzed the data separately for long (> 200 ms) and short (≥ 200 ms) ISIs.

As for long ISIs, we fitted linear models to the data. The null model consists of a single line, the respective alternative model contains two lines each of which represents one of the two respective conditions. For instance, the group effect was analyzed by the following two models:

(1) $$\text{alt}:y_{ijk}=\beta_{0}+\beta_{I}q_{i}+\beta_{G}\delta (j,\text{Chinese})+s_{k}+\varepsilon_{ijk}$$

(2) $$\text{null}:y_{ik}=\beta_{0}+\beta_{I}q_{i}+s_{k}+\varepsilon_{ik}$$

where *q_i_*: ISIs, *δ*(*x, y*) := 1 if *x *= *y*, 0 else, *s_k_*: random effects for interindividual differences in performance, and parameters *β *to be fitted, where *β*_0 _denotes the general slope and the other indices indicate the effect they represent (*I *for ISI, *G *for group). Unobserved stochastical components are denoted by *ε*.

Mask type effects were analyzed by analogous models:

$$\text{alt}:y_{ijk}=\beta_{0}+\beta_{I}q_{i}+\beta_{M}\delta (j,\text{scrambled})+s_{k}+\varepsilon_{ijk}$$

$$\text{null}:y_{ik}=\beta_{0}+\beta_{I}q_{i}+s_{k}+\varepsilon_{ik}.$$

As for short ISIs, the plots of the means of the expectation values indicate slight "U shapes" with a minimum for ISIs greater than zero, so that a linear model might not be a good choice. Therefore, we included a quadratic term (here for the group comparison):

(3) $$\text{alt}:y_{ijk}=\beta_{0}+\beta_{I}q_{i}+\beta_{\hat{I}}q_{i}^{2}+\beta_{G}\delta (j,\text{Chinese})+s_{k}+\varepsilon_{ijk}$$

(4) $$\text{null}:y_{ik}=\beta_{0}+\beta_{I}q_{i}+\beta_{\hat{I}}q_{i}^{2}+s_{k}+\varepsilon_{ik}$$

In comparison with linear models (1) and (2), the inclusion of quadratic terms, (3) and (4), yielded substantially better fits.

For the additional indicator models for Experiment 2 we did not separate long and short ISIs as we were interested in an overall effect. We fitted models with an additional quadratic term like in (3) and (4). Although models without the quadratic term yielded very similar results, the model fit for the quadratic models was substantially better.

#### Tests

The respective null and alternative models have been compared by two independent statistical tests. First, we calculated a likelihood ratio χ^2 ^test. This test computes a test statistics from the ratio of the likelihoods Λ for the null and the alternative model and approximates the test statistics -2 log Λ by a χ^2 ^distribution. The resulting *p *value is the probability, assuming the null is true, to obtain a value of the test statistic as or more extreme than the one obtained from our data. Note that this is not the probability that the null is true. This test was mainly chosen because it is conventional and frequently used for statistical model comparisons, and many readers will be familiar with it.

Second, we approximated the Bayes factors [25,27] by the Bayesian information criterion (BIC) [28], following Raftery, Eq. (29) [25] or Wagenmakers, Eq. (10) [26]:

$$\text{BF}\approx \text{exp}\left(\frac{\text{BIC(null)}–\text{BIC(alt)}}{\text{2}}\right).$$

## Authors' contributions

TE, CS, and JJ conceived of the study. TE and CS designed and programed the stimulus presentation software and the experiments. CS performed the data collection. TE and RS contributed to data analysis. All authors co-drafted the manuscript and accepted its final version.

## Supplementary Material

Additional file 1 **Supplementary Figures**. Additional figures showing the ratios of correct responses of the single observers. Click here for file
