# Supplementary material for: Chinese characters reveal impacts of prior experience on very early stages of perception
Source: BMC Neurosci. 2011 Jan 26;12:14. doi: 10.1186/1471-2202-12-14 (PMC3040141; doi:10.1186/1471-2202-12-14)
Supplement: Additional file 1 — Supplementary Figures. Additional figures showing the ratios of correct responses of the single observers. [file 1471-2202-12-14-S1.PDF]

Supplementary Figures

Supplements the article:

Chinese characters reveal impacts of prior experience on very early time course of perception

Authors: Tobias Elze, Chen Song, Rainer Stollhoff, Jürgen Jost

Figures S1 and S2 supplement Figures 4 and 5 from the article, respectively. While the figures in the article show means over expectation values, Figures S1 and S2 present the ratios of correct answers for each participant (pale lines). In addition, the averages over these ratios are plotted (triangles).

Note that averages over ratios of correct answers, although common in the literature, are not a useful measure for the central tendency of the data because of the underlying binomial distribution. We plot these averages only for the sake of comparability with other studies. For a more appropriate measure of central tendency please see the figures in the article.

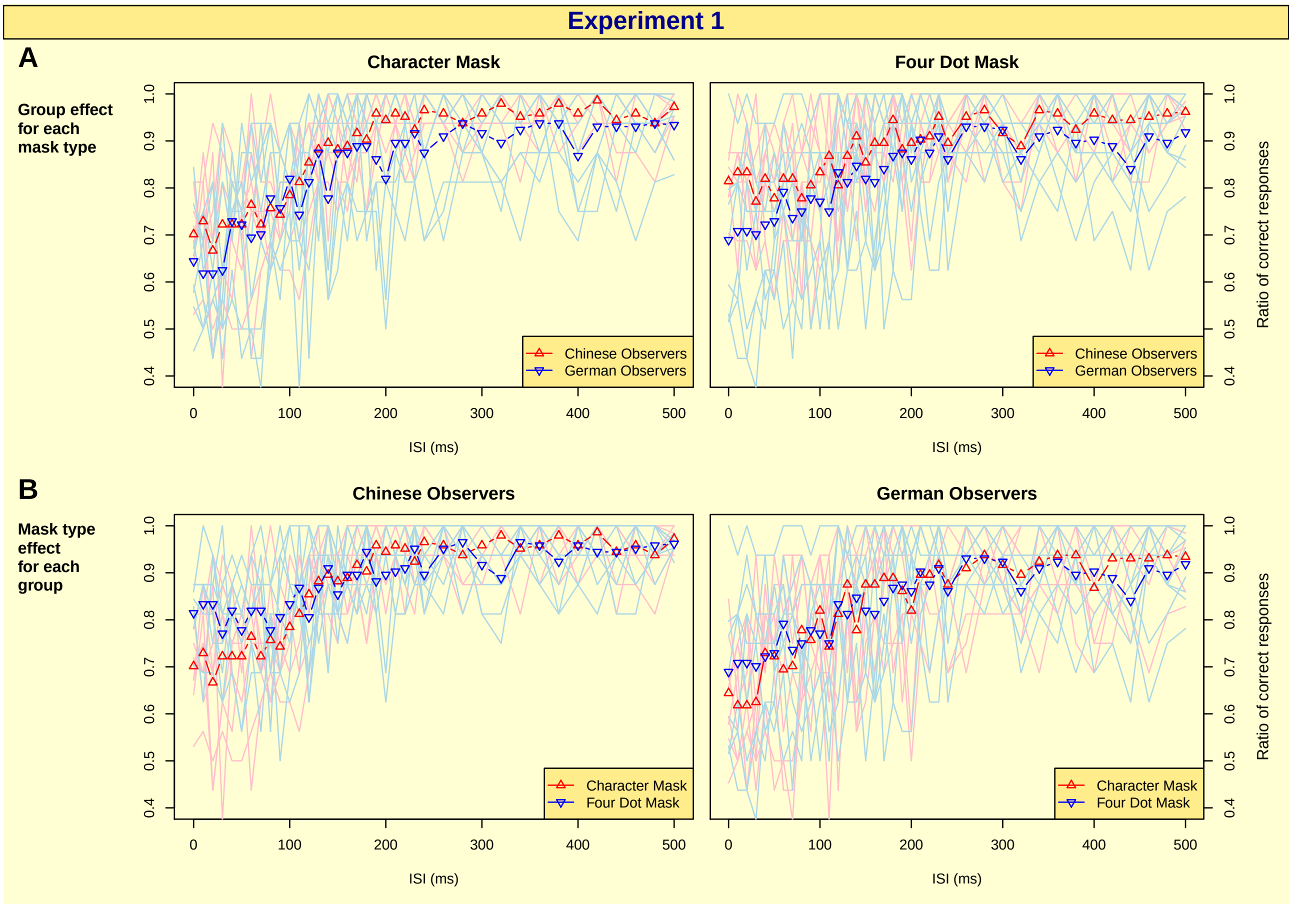

Figure S1: Ratios of correct responses in Exp. 1. The pale lines show the results of the single observers, the triangles the corresponding averages.

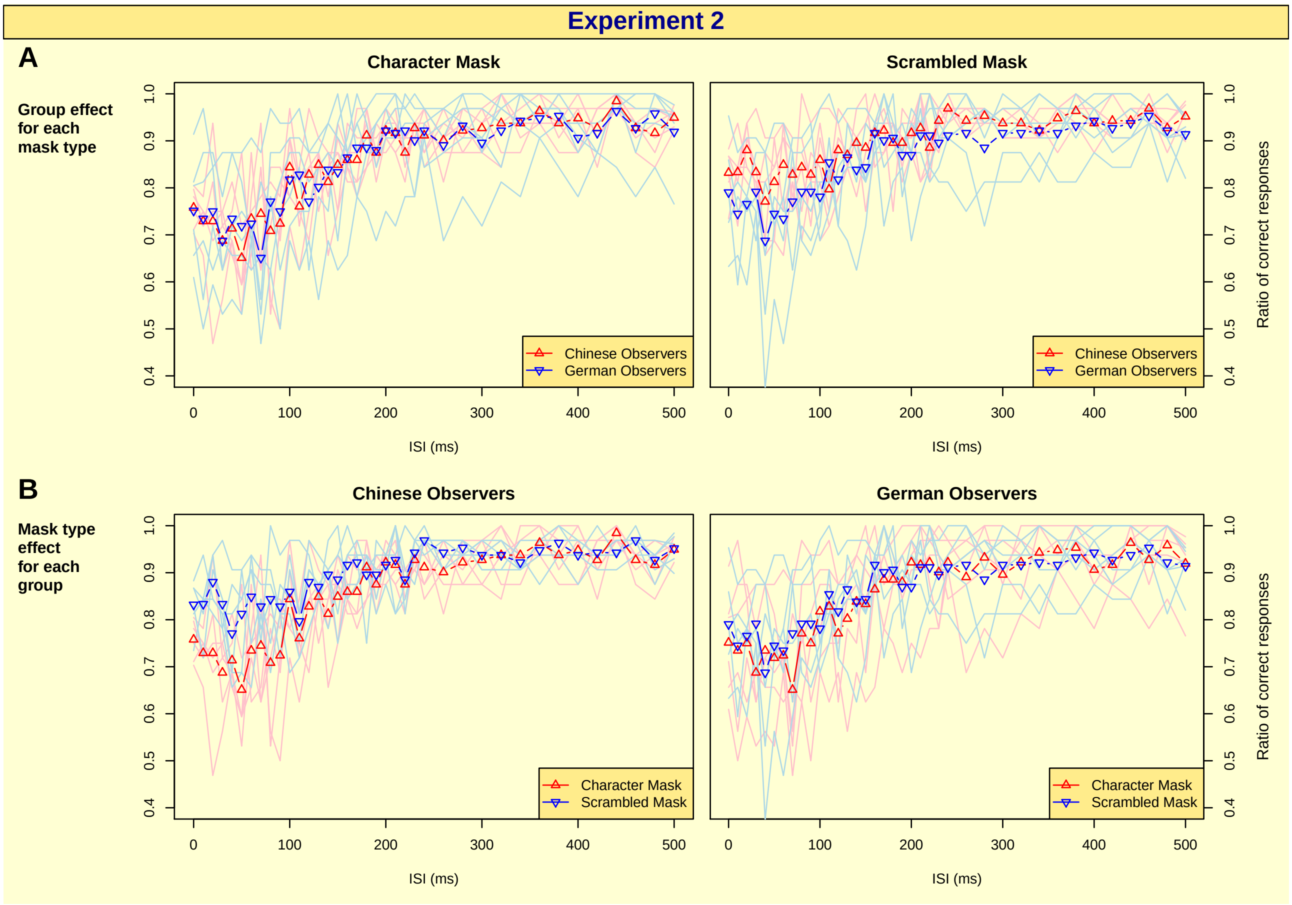

Figure S2: Ratios of correct responses in Exp. 2. The pale lines show the results of the single observers, the triangles the corresponding averages.
